# CDX-2, MUC-2 and B-catenin as intestinal markers in pure mucinous carcinoma of the breast

**DOI:** 10.1186/0717-6287-47-43

**Published:** 2014-09-18

**Authors:** Laura García-Labastida, Raquel Garza-Guajardo, Oralia Barboza-Quintana, Irám Pablo Rodríguez-Sanchez, Jesús Ancer-Rodríguez, Juan Pablo Flores-Gutierrez, Gabriela Sofía Gómez-Macías

**Affiliations:** Hospital Universitario “Dr. José Eleuterio González”, Departamento de Anatomía Patológica y Citopatología, Universidad Autónoma de Nuevo León, Madero and Gonzalitos S/N, Col. Mitras Centro, Monterrey, Nuevo Leon 64460 Mexico; Hospital Universitario “Dr. José Eleuterio González”, Departamento de Genética, Universidad Autónoma de Nuevo León, Av. Madero and Gonzalitos S/N, Col. Mitras Centro, Monterrey, Nuevo León 64460 Mexico

**Keywords:** Breast mucinous carcinoma, Inmunohistochemical, Intestinal differentiation

## Abstract

**Background:**

Pure mucinous adenocarcinoma of the breast is a rare entity characterized by the production of variable amounts of mucin comprising 1% to 6% of breast carcinomas. Some mucinous adenocarcinomas have shown expression of intestinal differentiation markers such as MUC-2. This study examines the expression of intestinal differentiation markers in this type of breast carcinoma.

**Results:**

Twenty-two cases of pure mucinous adenocarcinoma of the breast were assessed. Immunochemistry was performed for beta-catenin, CDX-2 and MUC-2. All cases were positive for B-catenin. MUC-2 positivity was observed in all cases; 63. 6% were 3 plus positive. All cases were negative for CDX-2.

**Conclusions:**

These results suggest that mucinous breast carcinomas express some markers of intestinal differentiation, such as MUC-2 and beta-catenin; however, future studies with a larger series of cases and using molecular techniques that help affirm these results are needed.

## Background

Pure mucinous adenocarcinoma of the breast (PMACB) is a rare entity characterized by the production of variable amounts of mucin. It comprises approximately 1% to 6% of breast carcinomas [1, 2]. Pure mucinous carcinomas are those with a mucinous component of more than 90% [3]. These tumors have a better prognosis than mixed or non-mucinous tumors [4, 5]. Mucin synthesis is a common feature of glandular tissue and has been studied in various adenocarcinomas. Multiple studies of PMACB have shown expression of the proteins MUC1, MUC2, MUC3, MUC4, MUC5A and MUC6, and these have been postulated as prognostic factors [6, 7]. The marker MUC2 is an intestinal-type mucin, which is mainly expressed in normal goblet cells of the colon and small intestine [8]. However, it is not only positive in colon cancer [9]. Its expression has also been shown in other mucinous carcinomas, which, despite their site of origin (salivary glands, pancreas, bladder, stomach, cervix, ovary, endometrium, and lung), share certain histological features. They are composed of glands lined with either columnar mucin producing cells with abundant extracellular mucin accumulation, or mucin-containing signet ring cells [10–12]. There are few published studies evaluating the expression of intestinal differentiation markers such as MUC2 in PMACB. Most compare their expression with usual ductal adenocarcinomas [6, 7, 13], and there are none that exclusively analyze more specific markers of intestinal differentiation, such as CDX-2 [14] and beta-catenin [15] in pure mucinous adenocarcinomas of the breast.

## Results

**Figure 1:**
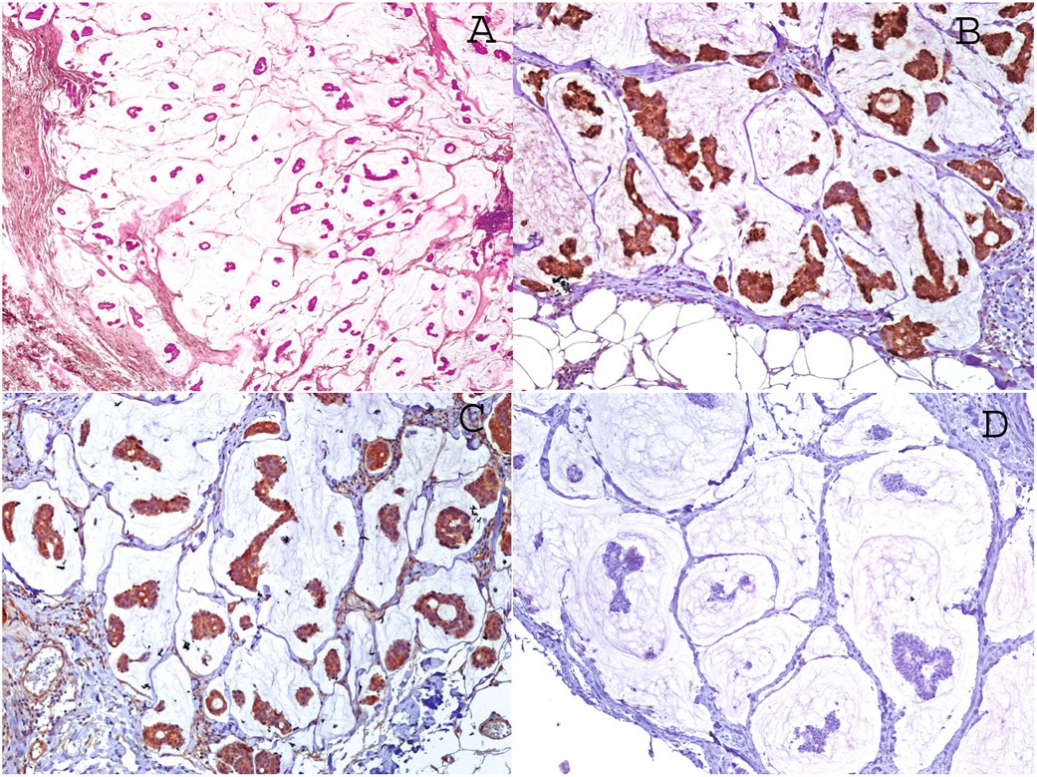
**Mucinous type adenocarcinoma of the breast, cases without DCIS. A.** A representative histopathological image H&E 5x. Immunohistochemical stains. **B.** MUC2 stain. **C.** Beta-catenin stain. **D.** CDX2 stain 10x.

**Figure 2:**
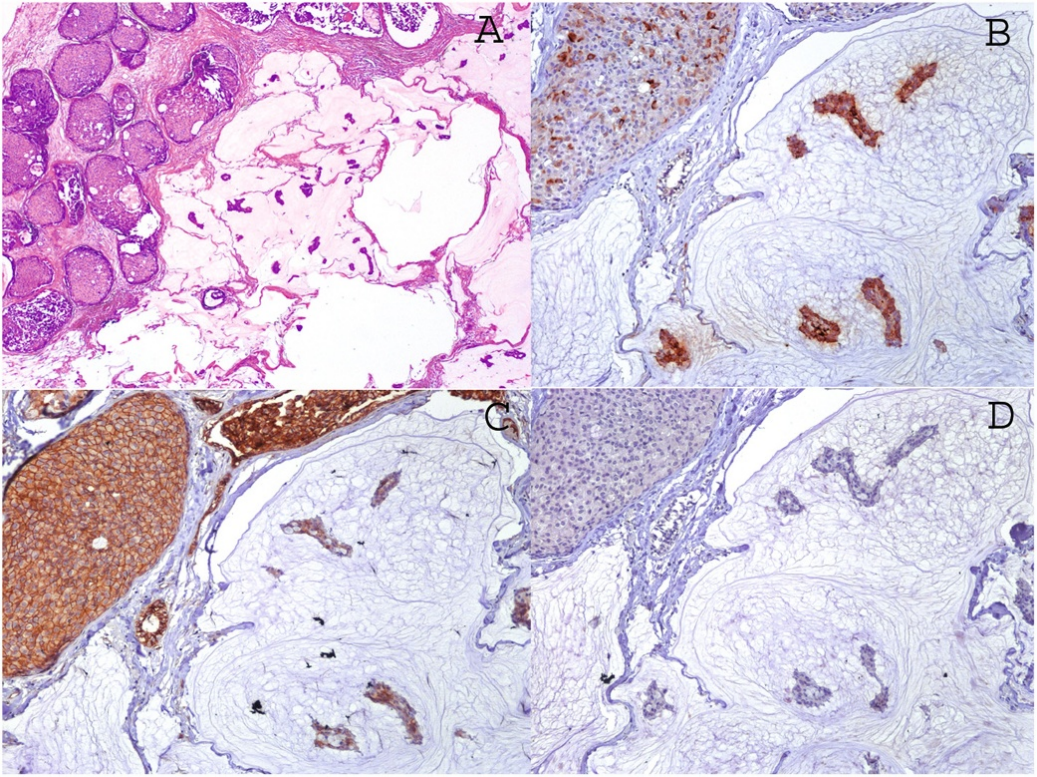
**Mucinous type adenocarcinoma of the breast, cases associated with DCIS. A.** A representative histopathological image H&E 5x. Immunohistochemical stains. **B.** MUC-2 stain. **C.** Beta-catenin stain. We interpreted the results as proportion 1 (10-25%) and weak intensity. **D.** CDX2 stain 10x.

During the period of 12 years there were 36 cases, of which only 22 (61.1%) were considered pure mucinous in their reassessment. Thirteen cases (59%) were associated with intraductal in situ carcinoma (DCIS). Mean age was 61 years (range 35–85) and mean tumor size was 2.49 cm (range 0.9-4.5 cm).All cases were positive for beta-catenin. The most frequently observed pattern (36.4%) was proportion 4 and strong intensity (>75% of cells). MUC2 positivity was observed in 100%; 63.6% were 3+ positive. Finally, all cases were negative for CDX-2 (Figures 1 and 2).

We compared the expression of the previously mentioned markers between those cases associated with in situ carcinoma and those without carcinoma in situ. This was done to evaluate the relationship between this histological parameter and the expression of intestinal differentiation markers. However, we found no statistically significant difference in any of the three markers (Table 1).

**Table 1:** **Comparison of expression of intestinal differentiation and the association with in situ carcinoma (DCIS)**

Marker	Without DCIS (n = 13)	With DCIS(n = 9)	*P*
**Intestinal differentiation markers, n (%)**			
Beta-catenin	13 (100)	9 (100)	.251
MUC 2	13 (100)	9 (100)	.298
CDX-2	0	0	Not applicable*

*No statistical value since all cases were negative.

## Discussion

Since pure mucinous adenocarcinoma of the breast (PMACB) is a variety of ductal carcinoma with specific histological features, it has attracted the interest of several authors for decades. Fujji et al. [16] demonstrated that PMACB possess a smaller number of genetic alterations in comparison with other variants of breast cancer. Lacroix-Triki et al. [17] found less genetic instability, suggesting that it is not only a histological entity, but also molecularly distinct from common ductal adenocarcinoma. Others studies, especially those from the late nineties, present the neuroendocrine differentiation of these neoplasms, which can occur in a variable percentage from 21% to 42% with histochemical studies, immunohistochemistry, and ultrastrcture [18–20]. The importance of this variety of breast adenocarcinoma is that numerous studies have shown that PMACB has a better prognosis than mixed or common ductal types [4, 5]. Volkan Adsay et al. [13] propose that secretion of distinct mucin, specifically to the stromal surface, acts as a container for neoplastic cells, reducing their ability to disseminate. The findings of this study may also explain why PMACB has a better prognosis than common ductal carcinomas.

Another study demonstrated that PMACB secretes an acetylated sialomucin, which is also found in mucinous carcinomas of the colon and stomach [21]. Furthermore, immunohistochemical studies show that in PMACB, the predominant molecule is MUC2 [6, 7, 13], which is negative in normal breast tissue. MUC2 and also, other intestinal secretory mucins [8, 9], have been found in other mucinous adenocarcinomas, such as those of the pancreas [22], salivary glands [23], biliary tract, the ampulla of Vater [24], the stomach [25], endometrium [26], lung [27], and ovary [28], among others.

Expression of MUC proteins in breast adenocarcinoma has been proposed as a prognostic factor. Specifically, the MUC2 marker in common ductal adenocarcinoma has been associated with less aggressive behavior, showing an inverse relationship with the presence of vascular invasion and lymph node metastasis [6, 7].

Regarding beta-catenin, this is a cell adhesion molecule that plays an important role in the Wnt signaling pathway. When the Wnt beta-catenin pathway is activated, it is translocated from the cell membrane to the cytoplasm and the nucleus where it interacts with genes that activate transcription factors [29]. Many studies have demonstrated activation of this pathway in adenocarcinoma of the breast [30, 31]. Also, it plays an important role in colorectal carcinogenesis, activating the APC gene pathway or the Wnt signaling pathway [10]. In cases of colorectal carcinoma, 90% show nuclear positivity for beta-catenin [32]. Although both adenocarcinomas share this molecular pathway, we could not find any case of pure mucinous adenocarcinoma of the breast that displays a nuclear staining pattern similar to that of colon adenocarcinoma. This agrees with the work by Peiguo Chu et al., who tried to determine the site of origin of 175 cases of mucinous adenocarcinoma. The authors included 18 cases that were found to be primary breast tumors and none showed nuclear positivity for beta-catenin [10]. Regardless of this, the majority of studies of beta-catenin expression in adenocarcinoma of the breast have tried to correlate its expression with clinical behavior. Decreased expression or aberrant expression has been associated with increased frequency of positive lymph node metastasis, and overexpression of the HER2 neu and basal phenotype [30, 31].

On the other hand, CDX2 is a gene belonging to the homeobox gene family, which is required for intestinal organogenesis and encoding nuclear transcription factors involved in the proliferation and differentiation of intestinal epithelium. The highest frequency of CDX2 staining occurred in colorectal carcinomas when compared with tumors from other sites. Therefore, the high frequency of CDX2 staining in colorectal adenocarcinomas, staining in extraintestinal tumors with intestinal-type epithelium, and uncommon staining in tumors from various sites lacking an intestinal phenotype sugg4ests that it is a useful marker for intestinal-type differentiation [14, 33]. O’Connell et al. [34] conducted a study of the role of immunohistochemistry in distinguishing primary adenocarcinomas of the gastrointestinal tract versus metastatic breast cancers, in which 47 adenocarcinomas involving the gastrointestinal tract were examined, including 19 cases of primary breast cancer. In their results, 100% of the cases from breast were negative for CDX-2. The results of our work and that of O’Connell are consistent with other studies in which, despite constant expression of MUC 2 in PMACB, no case showed positivity for CDX-2 [10]. However, among colorectal adenocarcinomas, the relationship between tumor grade and CDX2 staining has been controversial. CDX2 does not appear to be a sensitive marker for poorly differentiated colorectal carcinomas, and it is not a completely specific marker. Finally, CDX2 also stains other non-intestinal mucinous tumors, most notably carcinomas of lung, ovary and endometrium [14]. This can be a 'pitfall’ in assessing primary site or of a metastatic mucinous tumor of unknown origin, especially if it is poorly differentiated.

Although expression of these markers in other studies has been associated with prognosis and other histological parameters, we found no statistically significant differences between those cases with and without DCIS.

## Conclusions

In conclusion the results of this study suggest that mucinous breast carcinomas express some markers of intestinal differentiation; however, future studies containing a larger series and other molecular techniques that help affirm the results of this study are required.

## Methods

We performed a retrospective observational review of pathological reports of patients with mucinous type adenocarcinomas of the breast obtained from the Pathology and Cytopathology Department of the UANL University Hospital in Monterrey, Mexico in a period of 12 years (January 2000 to December 2011). We included only cases with at least 90% mucinous differentiation. We excluded those cases that did not have a paraffin block. All cases were reevaluated with the usual technique of hematoxylin and eosin, in 3 micron slices. The most representative slice of each tumor was selected and immunohistochemical staining was performed for beta-catenin, CDX-2 and MUC2. The immunohistochemical technique applied was based on the manufacturer’s recommendations for each antibody. The antibodies, company, dilutions and criteria used to evaluate positivity are summarized in Table 2. These were based on previous studies with the markers [6, 10, 29]. We included positive and negative controls for each marker on each slide. For statistical analysis, we used SPSS version 17.

**Table 2:** **Intestinal differentiation markers and parameters used to evaluate positivity**

Marker	Mark	Dilution	Positivity	Proportion	Intensity
MUC 2	Cell Marque MRQ-18	Prediluted	Luminar/Cytoplasmic Cytoplasmic/membranous	>5% of neoplastic cells	0 = Negative
					1 = weak
					2 = moderate
					3 = strong
CDX 2	Biocare Medical CDX2-88	1: 50	Nuclear	25% = 1+	1 + = weak
				26-75% = 2+	2 + = strong
				>75% = 3+	
B-catenin	Santa Cruz E-5	1: 50	Membranous	0 = 0 – 10%	0 = Negative
				1 = 10 – 25%	1 = weak
				2 = 25 – 50%	2 = moderate
				3 = 50 – 75%	3 = strong
				4 = >75%	

## Abbreviations

PMACB: Pure mucinous adenocarcinoma of the breast
ER: Estrogen receptor
PR: Progesterone receptor.
